# Obliterative portal venopathy: A neglected and probably misdiagnosed disease with peculiar etiology in South America

**DOI:** 10.1002/jgh3.12840

**Published:** 2022-11-16

**Authors:** Vinícius Nunes, Luiz A R de Freitas, Juliana R de Freitas, Caio Araújo, Gildásio N Junior, Maria I Schinoni, Fernando Bessone, Raymundo Paraná

**Affiliations:** ^1^ Gastroenterology and Hepatology Department Hospital Universitário Prof Edgard Santos Salvador Brazil; ^2^ Medical School of the Federal University of Bahia‐Brasil Salvador Brazil; ^3^ IDOR São Paulo Brazil; ^4^ Department of Pathology School of Medicine of the Federal University of Bahia Salvador Brazil; ^5^ LPEM of the Instituto de Pesquisa Gonçalo Moniz‐FIOCRUZ Salvador Brazil; ^6^ Faculty of Medicine Medical School of the Federal University of Bahia Salvador Brazil; ^7^ Hospital Provincial del Centenario University of Rosario School of Medicine Rosario Argentina

**Keywords:** drug‐induced liver injury, hepatoportal sclerosis, non‐cirrhotic portal hypertension

## Abstract

**Background and Aim:**

Obliterative portal venopathy (OPV) is one of the causes of non‐cirrhotic portal hypertension. However, many aspects of OPV remain unclear, including the etiology, pathogenesis, and natural history. The aim of this study was to describe the clinical features of OPV in a series of patients in Brazil in whom OPV was diagnosed through liver biopsy.

**Methods:**

Forty‐three consecutive adult patients with OPV were retrospectively selected as a case series based on histologic criteria, defined by the presence of at least portal fibrosis, phlebosclerosis, disappearance and/or reduction of the caliber of portal vein branches, and exclusion of cirrhosis. Clinical and laboratory data were analyzed. Clinically significant portal hypertension was considered in the presence of esophageal varices and/or ascites.

**Results:**

The mean age of patients at diagnosis was 44.5 ± 11 years, who were predominantly female (81%). Clinically significant portal hypertension was found in 28% of cases. The most frequent indication for liver biopsy was the elevation of liver enzymes, mostly γ‐glutamyl transferase (GGT) in 76% of patients, averaging 222 IU/L (upper limit of normality up to 40 IU/L) and alanine aminotransferase (ALT) in 64%, mean 84 IU/L (38 IU/L). One‐third of our patients had exposure to medications, especially herbal medicines, at the time of enzymatic changes. Other risk factors highlighted were features of autoimmunity in 25% of patients or thrombophilia in 20%.

**Conclusion:**

OPV can be diagnosed even before the onset of portal hypertension, ALT elevation, and especially GGT elevation in most cases. Its etiology is not defined, but autoimmune diseases, thrombophilia, and the use of medications or herbal medicines may play a role.

## Graphical abstract

Classically described in the group of non‐cirrhotic portal hypertension, obliterative portal venopathy (OPV) causes are still unknown. Our aim is to describe the characteristics of 43 OPV patients and potential risk factors. Clinically significant portal hypertension was found in 28% of cases. The most frequent indication for liver biopsy was elevation of liver enzymes, mostly γ‐glutamyl transferase increase in 76% of patients. One third of our patients have exposure to medications, especially herbal medicines at the time of enzymatic changes.

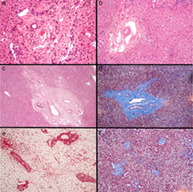

## Introduction

One disease with many names and no well‐established etiology, namely obliterative portal venopathy (OPV), often still described as idiopathic obliterative portal venopathy (IOPV), is one of the most neglected and underdiagnosed liver diseases. Diagnosis is dependent on histologic analysis of the liver and exclusion of other causes of non‐cirrhotic portal hypertension, mainly the hepatosplenic form of schistosomiasis. OPV is considered a rare vascular disease associated with presinusoidal portal hypertension.^1^, ^2^

OPV has also been termed non‐cirrhotic idiopathic portal hypertension, non‐cirrhotic portal fibrosis, porto‐sinusoidal vascular liver disease, and hepatoportal sclerosis. These designations have been coined over time in attempts to standardize relevant histologic findings. The most characteristic histopathologic finding is a fibrous enlargement of the portal tracts associated with either the disappearance or the reduction in the caliber of the portal vein branches in the absence of cirrhosis. Additional findings may include hypertrophy of the intrahepatic artery branches, anomalous lymphatic and/or venous vessels with telangiectatic appearance, paraportal herniated vessels, sinusoidal dilation and/or perisinusoidal fibrosis, and nodular regenerative hyperplasia of the hepatocytes.^2^, ^3^, ^4^

In northeastern Brazil, schistosomiasis is endemic; the hepatosplenic form remains a common cause of non‐cirrhotic portal hypertension. Accordingly, cases of OPV may be misdiagnosed as portal hypertension associated with schistosomiasis. These diseases share common clinical manifestations and histologic findings, with the exception of the presence of parasites in the latter.^5^

In OPV, signs of portal hypertension, such as large splenomegaly and esophageal varices, are common findings; however, ascites is rare, except in decompensated cases. Disease presentation is spectral, and diagnosis can be made prior to the onset of portal hypertension. However, the establishment of early diagnosis is challenging in clinical practice, as this is dependent on liver biopsy.^3^, ^6^

Historically, OPV was thought to be associated with several clinical conditions: recurrent gastrointestinal infections, HIV infection, autoimmune diseases,^7^ Adams–Olivier syndrome, a state of hypercoagulability (genetic thrombophilia or myeloproliferative diseases),^8^ primary immunodeficiencies,^9^ and medications, notably those derived from thiopurine such as azathioprine,^10^, ^11^ as well as vitamin A^12^ and other herbal and dietary supplements commonly taken in Bahia‐Brazil.^13^

The consumption of dietary supplements and/or herbal medicines has notably increased in recent decades.^14^

The use of herbal products and teas is extremely common in the Brazilian population, especially in the northeast, whose populations are greatly influenced by indigenous and African miscegenation.^15^

This study aimed to retrospectively evaluate the clinical and laboratory characteristics of 43 patients consecutively diagnosed with OPV. Possible risk factors for the disease were also analyzed, with emphasis placed on combinations of herbal and allopathic medicines with respect to the development of liver injury.

## Materials and methods

One‐hundred and eight patients with OPV were selected based on the histopathologic findings in a database from our pathology department from 1992 to 2019. The suspected diagnosis was based either on the typical pathologic findings description (Table 1) or on the presence of one of its synonyms written as a conclusion in the pathologist's report. Exclusion criteria for this analysis were patients under 18 years of age, those with associated background of a congenital liver disease, previous diagnosis, or exposure to schistosomes. For excluding schistosomiasis, we used major and minor criteria. The major ones were epidemiologic history, histopathology with no viable eggs, mummified eggs, or granuloma, and Kato Katz fecal exam negative. The minor ones were cutaneous or serology negative test. Samples from liver explants were not considered. Patients with previous or associated liver disease, such as viral hepatitis, autoimmune disease, alcohol or metabolic liver diseases, were also excluded. In addition, patients for whom the clinical and laboratory records were incomplete or not available were also ruled out.

**Table 1:** Frequency of histopathologic alterations found in liver samples from patients diagnosed with OPV

	OPV with portal hypertension (*n*, %)	OPV without portal hypertension (*n*, %)	*P*‐value
Number of biopsies	26 (100)	13 (100)	—
Type of biopsy			
Surgical (wedge)	25 (96)	11 (85)	—
Needle (percutaneous)	1 (4)	2 (15)	
Portal fibrosis	26 (100)	13 (100)	
Mild	6 (23)	3 (23)	—
Moderate	16 (61)	7 (54)	0.735
Severe	4 (16)	3 (23)	
Periductal fibrosis	19 (73)	8 (61)	0.486
Perisinusoidal fibrosis	22 (85)	8 (61)	0.129
Septal fibrosis	11 (42)	6 (46)	0.819
Portal vein disappearance	26 (100)	13 (100)	—
Portal vein diameter reduction	26 (100)	13 (100)	—
Portal vein herniation	8 (31)	5 (38)	0.725
Telangiectasis	26 (100)	13 (100)	—
Portal inflammation	12 (46)	2 (15)	0.083
Mild	10 (38)	2 (15)	
Moderate	2 (8)	0	0.548
Hypertrophy of intra‐hepatica arterial branches	18 (69)	7 (54)	0.482
Sinusoidal dilatation	10 (38)	6 (46)	0.645
Areas of parenchyma extinction	23 (88)	8 (61)	0.05
Regenerative nodular hyperplasia	14 (54)	3 (23)	0.068

The information obtained in medical records includes clinical variables (such as the time of initiation of medications, comorbidities, symptoms) and biochemical variables (such as liver enzyme levels) and liver function evaluation. Imaging and endoscopic characteristics were also evaluated for signs of clinically significant portal hypertension such as the formation of esophageal varices or collateral varices assessed by contrast CT scan and doppler ultrasound: patients with at least one of these findings were considered to have portal hypertension.

We retrospectively studied a series of 43 patients from a referral service for liver diseases, who were selected based on histologic disease criteria^3^ (Table 1). Only liver specimens larger than 1 cm in length and containing more than 12 portal tracts were considered for analysis. Most liver histology results were obtained by wedge biopsy through a diagnostic laparoscopic study. This kind of biopsy is preferred in our center in cases of suspected vascular diseases because it is more representative in terms of liver parenchyma compared to transcutaneous needle biopsy. Hematoxylin and eosin, Gomori reticulin stain, Perls for assessement of iron overload, and picrosirius red staining for fibrosis were used. All cases were evaluated and reviewed by a pathologist experienced in liver pathology.

Statistical analysis was performed using the SPSS program, version 16. Discrete variables were described as frequencies, and continuous variables as descriptive statistics via means and intervals.

This project was approved by the institutional review board of the Gonçalo Moniz Institute (IGM‐FIOCRUZ‐Bahia, protocol number: 757,935).

## Results

The mean age of patients at the time of biopsy was 44.5 years (range 18–72 years). There was a predominance of females (81%). Clinically significant portal hypertension was found in 28% of the patients, while ascites or portal vein thrombosis was identified in only three and two patients, respectively.

Persistent abnormalities in liver biochemistry were the main indications for liver biopsy, with the values shown in Table 2 representative of those most proximate to the time of the biopsy. Regarding liver function, all patients had a prothrombin time above 70% (minimum value: 72%, mean 95%). The highest bilirubin level observed was 1.7 mg/dL (mean: 0.7 mg/dL). Mean albumin was 4.1 g/dL (SD 0.5) and the only two patients who presented values below 3.5 g/dL (3.2 and 2.2 g/dL) also had ascites.

**Table 2:** Demographic and laboratory data from 43 patients diagnosed with OPV

Demographic variables			
Mean age	44.5 years		
Sex (male/female)	8 (19%)/35 (81%)		
Portal hypertension	12 (28%)		

Laboratory variables	Values	% (*N*)	Mean (U/L)
ALT	<1 ULN	36 (15)	84
	1–2 ULN	22 (9)	
	>2 ULN	42 (17)	
AST	<1 ULN	42 (17)	57
	1–2 ULN	37 (15)	
	>2 ULN	21 (9)	
ALP	<1 ULN	51 (21)	168
	1–2 ULN	28 (12)	
	>2 ULN	21 (8)	
GGT	<1 ULN	24 (10)	222
	1–2 ULN	10 (5)	
	>2 ULN	66 (26)	
Platelets/mm^3^	<150 000	22 (8)	206.281
	>150 000	78 (30)	

ALP, alkaline phosphatase; ALT, alanine aminotransferase; AST, aspartate aminotransferase; GGT, γ‐glutamyl transferase; ULN, upper limit of normality.

**Figure 1:**
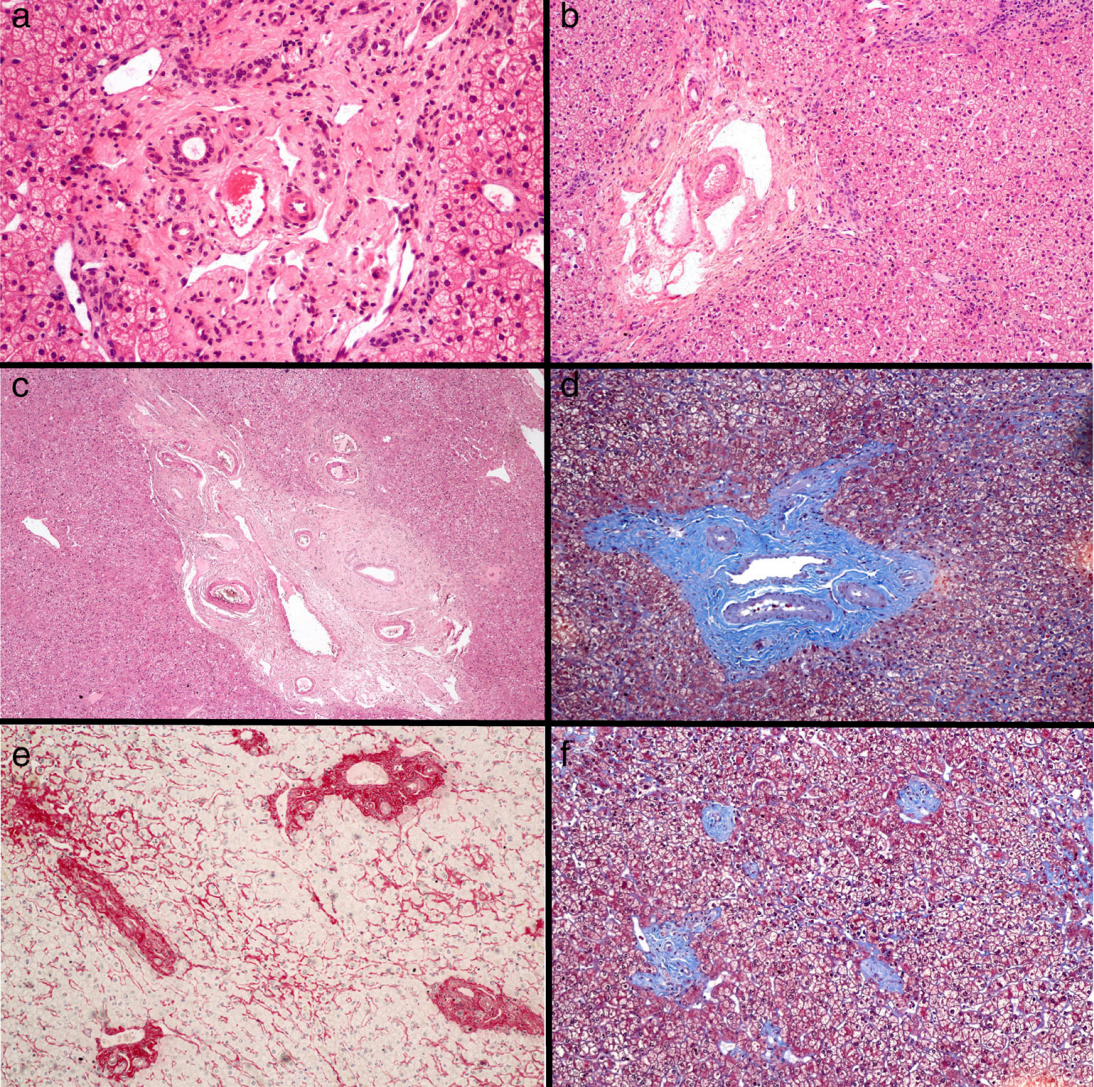
(a) Portal tract enlarged by fibrosis with marked reduction in the caliber of the portal vein branch, presence of telangiectatic vessels, and paraportal vessels (H&E ×100). (b) Portal tract with fibrosis, showing anomalous venous vessels, with vessel margination (H&E ×10). (c) Portal fibrosis, reduction in the caliber of the branch of the portal vein, arterial hypertrophy, and periductal fibrosis (H&E ×10). (d) Portal fibrosis, reduced caliber of the portal vein, arterial hypertrophy, and short septum. Discrete perisinusoidal fibrosis is observed (Masson's trichrome, ×10). (e) Approximation of portal tracts with veins of reduced caliber, margination of portal vein branches, and clear perisinusoidal fibrosis (Picro‐sirius red, ×4). (f) Approximation of atrophic portal spaces, without portal vein branches (parenchyma extinction) (Trichromic from Masson, ×4).

The histopathologic findings of 39 patients are summarized in Table 1; Figure 1 depicts some characteristic histopathologic alterations of OPV.

The risk factors associated with OPV identified in the present sample are listed in Table 3. At least one of the risk factors was present in 77% of patients, and 18% presented more than one risk factor. No evidence suggestive of other liver diseases was detected in the patients.

**Table 3:** Risk factors possibly associated with OPV

Risk factor	% (*n*)	Description	*N*
Use of drugs and/or herbal products^†^	32 (19)	Herbs and dietary supplements: Herbalife products	6
		Vitamin A	1
		Oxandrolone	1
		*Uncaria tomentosa*	1
		*Equisetum arvense*	1
		*Monteverdia ilicifolia*	1
		Hormones (replacement therapy)	4
		Oxaliplatin	2
		NSAIDs	2
		Methotrexate	1
		Benzene	1
Autoimmunity	25 (11)	Autoantibodies: ANA ≥160	6
		ASMA ≥80	1
		Thyroiditis	3
		Rheumatoid arthritis	1
		Systemic Erythematosus Lupus	1
Thrombophilia	20 (09)	Factor V Leiden mutation	2
		Antithrombin deficiency	1
		Antiphospholipid syndrome	2
		Mthfr mutation	2^‡^
		Prothrombin mutation	1
		Protein C deficiency	1

^†^ Some patients used more than one drug/product.

^‡^ Absence of hyperhomocysteinemia.

ANA, antinuclear factor antibody; ASMA, anti‐smooth muscle antibody; MTHFR, methylenetetrahydrofolate reductase; NSAID, non‐steroidal anti‐inflammatory drug.

## Discussion

OPV is a cause of presinusoidal intrahepatic portal hypertension. Although classified in the group of non‐cirrhotic portal hypertensions, this disease is spectral, and portal hypertension is not always present. Previous studies have demonstrated that diagnosis can be made in the initial phase of the disease, prior to the development of portal hypertension.^3^, ^6^, ^9^ In fact, not all patients develop portal hypertension during follow‐up. In our series, less than one‐third of patients presented significant portal hypertension.

Portal vein thrombosis may be present in up to 30–40% of OPV patients, especially in association with more advanced portal hypertension, either as a cause or as a consequence of it. In many older reports involving cohorts, diagnosis was achieved only following the appearance of clinical complications, such as the presence of ascites or episodes of gastrointestinal hemorrhage.^16^, ^17^, ^18^, ^19^ In our series, which included patients primarily diagnosed following biopsy because of persistent biochemical abnormalities, only 6% and 4% presented portal vein thrombosis or ascites, respectively. Perhaps early diagnosis due to the detection of biochemical alterations explains the low frequencies of portal hypertension and thrombosis in our sample.

Brazil accounts for 96% of all schistosome cases occurring in Latin America, and northeastern Brazil, where most of these cases are identified, is endemic for *Schistosoma mansoni*. In some cities, the prevalence of *S. mansoni* infection has reached over 15% of the local population.^5^ Fortunately, the severe form, termed hepatosplenic schistosomiasis, accounts for no more than 10% of all infected individuals. Severe disease development is characterized by presinusoidal portal hypertension consequent to the deposition of numerous schistosome eggs along the portal vein branches, which generates chronic granulomatous inflammation leading to fibrous expansion of the portal tract and intrahepatic portal vein obstruction.

In addition, less severe forms, such as intestinal schistosomiasis, develop without liver involvement. The hepatointestinal form includes liver involvement, yet in the absence of portal hypertension; portal fibrosis is an associated finding due to schistosome egg deposition in portal vein branches.^20^

The morphological alterations seen in the liver in schistosomiasis can histologically resemble OPV: that is, arterial hypertrophy, parenchymal atrophy, and periportal or perisinusoidal fibrosis may be present in both. However, positive serology and traces of parasite eggs and granulomas in portal hepatic tissue are distinct to schistosomal infection.^20^, ^21^ As a consequence, until recently, in areas endemic for schistosomiasis in Brazil, almost all cases of non‐cirrhotic portal hypertension, even those not biopsied, were attributed to *S. mansoni* infection.^22^

We were motivated to revisit this topic not only due to the potential occurrence of misdiagnosis in the past but also given the reduction in cases of the hepatosplenic form over the last two decades, as well as the fact that many patients do not reside in endemic areas. In addition, our liver unit has frequently diagnosed cases of OPV, mainly in patients with persistent abnormal liver enzymes. Our analysis further leads us to believe that patients with OPV could have been misdiagnosed with schistosomiasis in the recent past.^4^

While the causes underlying OPV remain unclear, some authors have described strong associations with procoagulant and immunological conditions, as well as genetic predisposition.^23^ Another possible association with drugs has been described in several case reports.^10^ The contribution of this study is to demonstrate the frequency of use of allopathic medicine and/or herbal/dietary supplements in patients with OPV in Latin America.

Attention should be paid to the fact that almost one‐third of our patients reported exposure to medications, especially herbal products, at the time when enzymatic changes were detected, which was often the indication for liver biopsy. While the use of herbal products has been rarely reported in other previously published series, associations between OPV and exposure to xenobiotics, such as thiopurine derivatives, didanosine, arsenic, busulfan, methotrexate, oxaliplatin, or high doses of vitamin A, were explored.^1^, ^10^, ^11^ Our results serve to solidify the notion of a toxic contaminant being involved in disease pathogenesis, as was reported by other authors, including two previously published reports on patients also included in the present series.^13^

Typical hepatotoxicity (i.e. elevated aminotransferase five times the normal upper limit or alkaline phosphatase twice the limit) associated with the use of herbs and dietary supplements represents 8% (29 cases) of all cases of hepatotoxicity reported in Latin America,^14^ reaching 21% in the local epidemiologic setting of the state of Bahia–Brazil.^24^ Moreover, liver damage may extend beyond classical inflammatory/cholestatic injury due to the presence of other substances, which may theoretically be linked to prothrombotic or autoimmune events, which therefore act as an OPV trigger. Endothelial injury and thrombosis have been described in association with pyrrolizidine alkaloids, often found in plant extracts.^25^, ^26^

The relationship between the consumption of dietary/herbal supplements, whose formulation is unverifiable, and the development of disease in six of our patients should be highlighted. In one of these cases, an improvement in biochemical patterns was observed following the discontinuation of herbal supplements, which then worsened after the patient resumed their use.

The consumption of herbal products often occurs in combination with other herbs or allopathic medicines, impeding the determination of causality. As an example, both the patient who used *Uncaria tomentosa*, popularly known as “Unha de gato,” and another who used *Monteverdia ilicifolia* (Espinheira Santa) also took non‐steroidal inflammatory drugs simultaneously. It is worth noting that the plant‐based product that a patient believes he/she is making use of does not often correspond to the element truly being consumed. Furthermore, many herbal/dietary products may be adulterated or even contaminated.^15^ Another point of concern is that herbal products can interact with allopathic substances and potentiate toxicity in unpredictable ways and also induce endothelial damage.^27^, ^28^

Other studies reported procoagulant disorders occurring in 5–33% of the studied patients, which is consistent with the 20% rate found in our population. These include coagulation factor abnormalities,^18^, ^19^, ^29^ antiphospholipid antibody syndrome (SAAF), and myeloproliferative diseases,^2^, ^8^ as well as endothelial alterations.^30^

The liver biochemical abnormalities identified in our patients follow patterns described in previous series, with a predominance of patients presenting elevated liver enzymes, especially GGT, which was elevated in 76% of reported cases.^6^, ^16^ The presence of autoantibodies reached 39% in Asian and Western series, with 12% and 17% being associated autoimmune diseases, respectively.^3^, ^6^, ^7^ Compared to other non‐autoimmune liver diseases, autoantibodies are present in around 5% of OPV patients.^7^ In our sample, 22% presented autoantibodies, with antinuclear antibody (ANA) ≥1:80 in six patients, anticardiolipin in two, and anti‐smooth muscle and rheumatoid factor in a single patient.

Primary immunodeficiency, which was described as one of the main associated etiologies in an American cohort,^9^ is scarcely reported in other populations. The other autoimmune diseases cited in the literature are chronic thyroiditis,^7^ celiac disease, ulcerative colitis, multiple sclerosis, granulomatous glomerulonephritis, rheumatoid arthritis (RA), systemic lupus erythematosus (SLE), myasthenia gravis, Sjogren's syndrome and scleroderma, autoimmune hepatitis, and primary biliary cholangitis.^3^, ^6^, ^10^, ^31^ In our cohort, concomitant liver diseases were excluded, while SLE, RA, and chronic thyroiditis were the other autoimmune diseases identified. HIV infection is also directly associated with OPV, as is the use of antiretrovirals, especially didanosine, yet this pathology was not present in our sample.^32^

Finally, the frequent histopathologic finding of periductal fibrosis in our series is intriguing. None of the patients had a clinical or radiological suspicion of sclerosing cholangitis (SC) or any other obstructive cholestasis. It has been suggested that an injury in one of the components of the portal triad (artery, portal vein, or bile duct) may implicate the involvement of other components as well, a phenomenon described as “menage au foie.”^33^

The main limitation of our work is the retrospective nature of the case series; however, our findings shed important light on this intriguing disease etiology. A control case study, preferably multicentric, would represent a great contribution to the understanding of OPV risk in association with the consumption of xenobiotics.

## Conclusion

OPV can be diagnosed prior to the onset of portal hypertension. ALT and especially GGT elevation occur in most cases. While the etiology of this pathology has not been defined, autoimmune diseases, thrombophilia, and the use of medications or dietary/herbal products may play a role. In Latin America, especially in Brazil, OPV must be strongly considered as a differential diagnosis for schistosomiasis.
